# Pediatric adrenocortical carcinoma: clinical features and application of neoadjuvant chemotherapy

**DOI:** 10.1186/s40001-023-01381-3

**Published:** 2023-10-09

**Authors:** Yu Lin, Shen Yang, Wei Yang, Haiyan Cheng, Xiaofeng Chang, Zhiyun Zhu, Jun Feng, Jianyu Han, Qinghua Ren, Saishuo Chang, Shan Liu, Tong Yu, Boren Hou, Pengfei Li, Deguang Meng, Xianwei Zhang, Hong Qin, Huanmin Wang

**Affiliations:** 1 Department of Oncology Surgery, Beijing Children’s Hospital, National Center for Children’s Health, Capital Medical University, Beijing, 100045 China; 2 Medical Imaging Center, Beijing Children’s Hospital, National Center for Children’s Health, Capital Medical University, Beijing, 100045 China; 3 Department of Surgical Oncology, Baoding Children’s Hospital, Baoding, 071051 Hebei China; 4 Department of Pediatric Oncologic Surgery, Henan Children’s Hospital, Zhengzhou Children’s Hospital, Children’s Hospital Affiliated to Zhengzhou University, Zhengzhou, 450018 Henan China

**Keywords:** Children, Adrenal cortical carcinoma, Neoadjuvant chemotherapy, Clinical features, Prognosis

## Abstract

**Objective:**

To summarize the clinical characteristics of children with adrenocortical carcinoma (ACC) and preliminarily explore the indications for and efficacy of neoadjuvant chemotherapy in certain patients.

**Methods:**

The data of 49 children with adrenocortical tumors (ACT) in the past 15 years were retrospectively analyzed, and after pathology assessment using Weiss system grading, 40 children diagnosed with ACC were included. Response Evaluation Criteria in Solid Tumors (RECIST) 1.1 and three-dimensional (3D) reconstruction of contrast-enhanced computed tomography data were used to evaluate the response to neoadjuvant chemotherapy.

**Results:**

Forty patients (17 males, 23 females) with ACC were enrolled. Abnormal hormone levels were common in children with ACC (*n* = 31), and in terms of clinical presentation, sexual precocity was the most common (*n* = 14, 35.0%), followed by Cushing’s syndrome (*n* = 12, 30.0%). Seven of 40 children received neoadjuvant chemotherapy due to a maximum lesion diameter greater than 10 cm (*n* = 4), invasion of surrounding tissues (*n* = 2), intravenous tumor thrombus (*n* = 2), and/or distant metastasis (*n* = 2); 2 patients achieved partial response, and 5 had stable disease according to the RECIST 1.1 standard. Furthermore, 3D tumor volume reconstruction was performed in 5 children before and after neoadjuvant chemotherapy. Tumor volumes were significantly reduced in all 5 children, with a median volume reduction of 270 (interquartile range, IQR 83, 293) (range: 49–413) ml. After surgery with/without chemotherapy, the 5-year overall survival rate for all children was 90.0% (95% CI-confidence interval 80.0–100.0%), and the 5-year event-free survival rate was 81.5% (95% CI 68.0–97.7%).

**Conclusion:**

In the diagnosis and treatment of pediatric ACC, a comprehensive endocrine evaluation is necessary to facilitate early diagnosis. Surgery and chemotherapy are important components of ACC treatment, and neoadjuvant chemotherapy should be considered for children with ACC who meet certain criteria, such as a large tumor, distant metastases, or poor general condition.

## Introduction

Adrenal cortical carcinoma (ACC) is an aggressive malignant tumor with one of the incidence peaks in children and adolescents, with 0.5–2.0 new cases per 1 million children per year, accounting for 0.2% of childhood malignant tumors [1, 2]. Due to the rarity of ACC, research on the disease is still mainly based on small numbers of case reports [3]. Surgery is a critical component of ACC treatment, but a high risk of recurrence remains [4]. After comprehensive treatment, the 5-year overall survival (OS) of stage IV patients who were previously not considered for surgery is 16% [5, 6]. Therefore, exploring new treatment modalities may be of great significance for improving the prognosis of ACC. In our study, we aimed to summarize the clinical data of ACC patients with a clear pathological diagnosis and to explore the best indication for and efficacy of neoadjuvant chemotherapy.

## Patients and methods

### Patients

A retrospective analysis of 49 children with adrenocortical tumors (ACT) admitted to the Beijing Children’s Hospital, Henan Children’s Hospital, and Baoding Children’s Hospital between April 2007 and February 2022 was performed. The Weiss score was used to determine the benignity and malignancy of the tumor, including: nuclear anisotropy; nuclear division index ≥ 5/50 high power field (HPF); atypical nuclear division; clear cells ≤ 25% of all cells; diffuse distribution of tumor cells; tumor necrosis; venous invasion; sinusoidal infiltration; and capsule infiltration. The system assigns a score of 1 to each of the 9 histological criteria, and a score ≥ 3 is classified as malignant [7, 8]. After pathology assessment using Weiss system grading, we diagnosed 9 of 49 as adrenocortical adenoma (ACA) and excluded them from the analysis. The remaining 40 children diagnosed with ACC were included in our study. Clinical characteristics, laboratory examinations, treatment plans, and imaging examinations were collected retrospectively. The patients’ pathological tests were reviewed and verified by a senior pathologist.

Patients were staged according to the European Adrenal Tumor Research Network (ENSAT) staging system [9]. Before treatment, all children underwent a thorough evaluation to assess tumor stage and surgical resectability of the primary tumor via abdominal enhanced CT, cranial CT, chest CT and/or PET–CT. Response Evaluation Criteria in Solid Tumors (RECIST 1.1) [10] and three-dimensional (3D) reconstruction of enhanced computed tomography (CT) scans were used to evaluate the response to neoadjuvant chemotherapy. All the children in this study were followed up through outpatient reexamination and by telephone. The frequency of follow-up after treatment is quarterly in the first year, biannually in the second–third year, and annually until the child reaches maturity. This retrospective study was approved by the Medical Ethics Committee of the Beijing Children’s Hospital [2022]-E-211-R, and family/patient informed consent requirements were waived.

### Statistical methods

SPSS 26.0 was used for statistical processing. The variables were tested for normality; normally distributed measurement data are expressed as mean ± SD, and non-normally distributed measurement data are expressed as median (interquartile range, IQR) (range: min–max). Count data are described by percentage. Length of time when the child did not experience tumor recurrence/progression, death, or development of a second tumor during follow-up was defined as event-free survival (EFS). EFS and OS were analyzed by the Kaplan–Meier method and log-rank test, and survival curves were drawn. *p* < 0.05 was considered statistically significant.

## Results

### Clinical characteristics

The median age of onset was 41.5 (IQR 18.0, 86.7) (range: 3.0–139.0) months, with a median tumor size of 6.5 (IQR 5.9, 7.0) (range: 2.1–15.0) cm in 40 children with ACC. Slightly more female (*n* = 23, 57.5%) than male (*n* = 17, 42.5%) children were included. The tumors were all located in a unilateral adrenal gland; 20 cases originated in the left adrenal gland (*n* = 20, 50%). In terms of clinical presentation, sexual precocity was the most common (*n* = 14, 35.0%), followed by Cushing’s syndrome (*n* = 12, 30%; including hypertension, *n* = 3), nonspecific findings (palpable mass, abdominal pain, fever, bleached) (*n* = 7, 17.5%), found incidentally during examination (*n* = 6, 15%), and virilization (*n* = 5, 12.5%); primary aldosteronism was the least common (*n* = 1, 2.5%). Abnormal levels of hormones were common in children with ACC (*n* = 31), including serum testosterone (*n* = 17), progesterone (*n* = 17), estradiol (*n* = 11), cortisol (*n* = 10), follicle-stimulating hormone (*n* = 6), prolactin (*n* = 5), adrenocorticotropic hormone (*n* = 3), and luteinizing hormone (*n* = 1). More than half of these children had two or more types of hormone abnormalities (*n* = 23). According to the ENSAT staging system, there were 14 patients (35.0%) with stage I disease, 16 (40.0%) with stage II disease, 7 (17.5%) with stage III disease, and 3 (12.5%) with stage IV disease (1 patient had multiple metastases to the lungs, 1 patient had inferior vena cava thrombosis with upward extension to the entrance of the right atrium, and 1 patient developed skin, brain and subaxillary lymph node metastasis) (Table 1).

**Table 1:** Hormone levels at the time of initial diagnosis

Variables	Level	Number
Cortisol	High	10
	Normal	20
	Unknown	10
Adrenocorticotropic hormone	High	3
	Normal	27
	Unknown	10
Luteinizing hormone	High	1
	Normal	28
	Unknown	11
Follicle-stimulating hormone	High	6
	Normal	23
	Unknown	11
Serum testosterone	High	17
	Normal	11
	Unknown	12
Estradiol	High	11
	Normal	18
	Unknown	11
Prolactin	High	5
	Normal	24
	Unknown	11
Progesterone	High	17
	Normal	13
	Unknown	10

### Treatment methods

At the initial diagnosis, 33 patients received immediate surgical treatment. The remaining 7 patients underwent biopsy before receiving neoadjuvant chemotherapy regimens of EDP (etoposide, doxorubicin, cisplatin) with cyclophosphamide and subsequent surgical treatment. Seventeen patients received postoperative chemotherapy, identical to neoadjuvant chemotherapy. Six patients with ENSAT stage II disease were treated with postoperative chemotherapy (2 cases of tumor > 10 cm, 1 case of intraoperative tumor rupture, and 3 cases of pathological results showing tumor capsule infiltration and a high mitotic count).

### Neoadjuvant chemotherapy

Seven (stage IV, *n* = 3; stage III, *n* = 3; stage II, *n* = 1) of the 40 children with ACC received neoadjuvant chemotherapy because of a maximum diameter greater than 10 cm (*n* = 4), invasion of surrounding tissues (*n* = 2), intravenous tumor thrombus (*n* = 2), and distant metastasis (*n* = 2). Patients who received neoadjuvant chemotherapy had larger tumors at diagnosis than those who did not (all *p* < 0.0001, Table 2).

**Table 2:** Comparison between patients with and without neoadjuvant chemotherapy

Variables		Neoadjuvant chemotherapy (*n* = 7)	No neoadjuvant chemotherapy (*n* = 33)	*p*
Age (median, IQR, months)		43.0 (12.0, 77.0)	40.0 (18.0, 94.0)	0.8100
Tumor size (at diagnosis) (median, IQR, cm)		10.5 (7.8, 13.3)	5.6 (4.6,6.5)	< 0.0001
Hormonal disorder	Yes	5 (71.4)	26 (78.8)	–
	No	0	1 (3.0)	
	Missing data	2 (28.6)	6 (18.2)	
ENSAT stage	I	0	14	–
	II	1	15	
	III	3	4	
	IV	3	0	

**Fig. 1:**
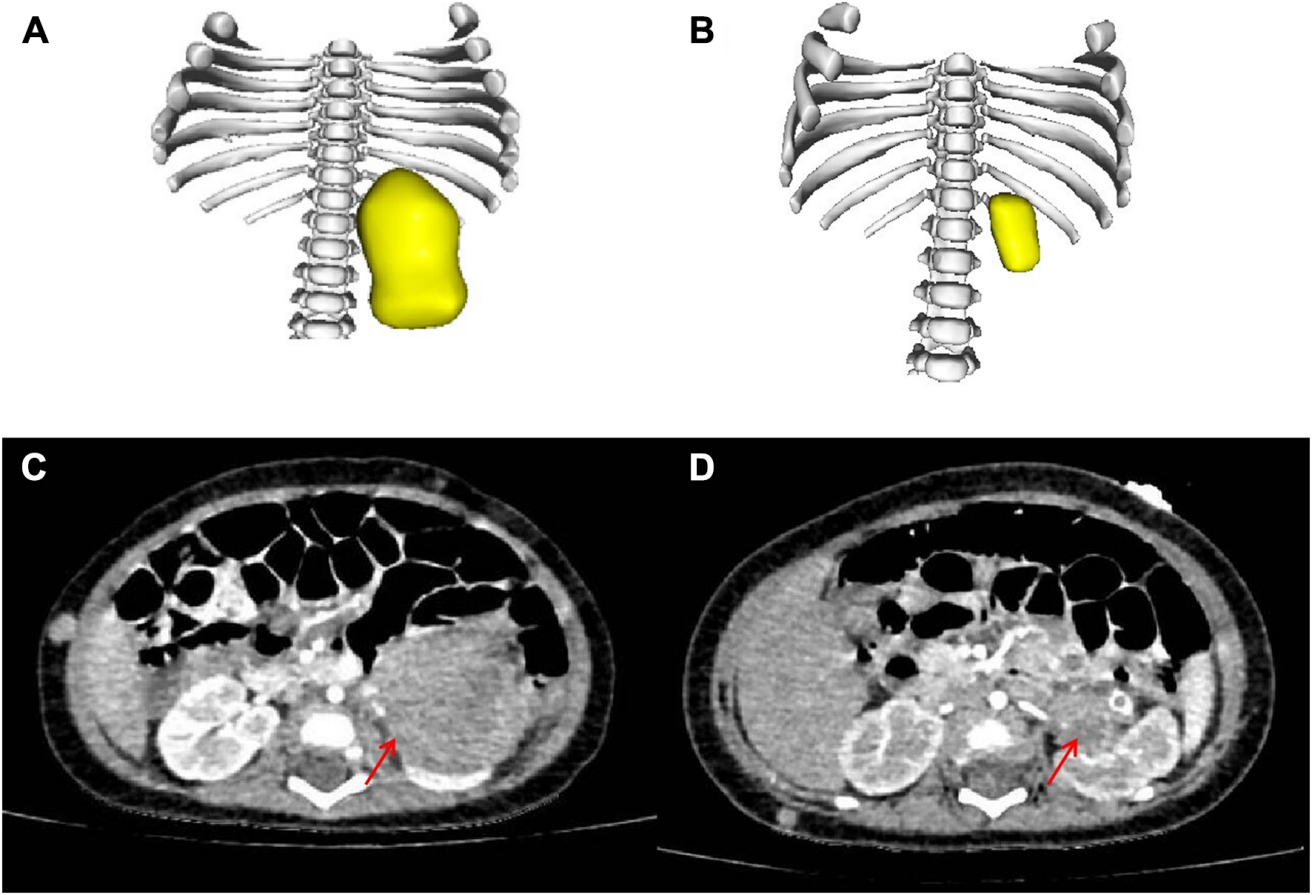
Evaluation of response to neoadjuvant chemotherapy in one patient with ACC. **A**, **B** 3D reconstruction before (**A**) and after (**B**) neoadjuvant chemotherapy in a 4-month-old patient with stage IV ACC. **C**, **D** Enhanced CT before (**C**) and after (**D**) neoadjuvant chemotherapy. Location of the primary tumor focus (arrow)

In terms of response evaluation, 5 patients had stable disease (SD), and 2 patients had partial remission (PR) according to the RECIST 1.1 standard. 3D tumor volume reconstruction was performed in 5 children before and after neoadjuvant chemotherapy (original image data from the initial enhanced CT examination were not available for 2 patients) [2]. The tumor volumes in all 5 children were significantly reduced, with a median volume reduction of 270 (IQR 83, 293) (range 49–413) ml; the most obvious reduction was from 55.7 to 6.6 ml (88.2%) (Table 3, Fig. 1). All 7 children received surgical treatment after neoadjuvant chemotherapy and received an additional 4–6 courses post-surgery.

**Table 3:** Clinical characteristics of 7 children who received neoadjuvant chemotherapy

Number	Age (m)	Stage	Maximum tumor size before neoadjuvant chemotherapy (cm)	Maximum tumor size after neoadjuvant chemotherapy (cm)	Change in maximum diameter (cm)	RECIST	Volume before neoadjuvant chemotherapy (ml)	Volume after neoadjuvant chemotherapy (ml)	Change in volume (ml)	Percent reduction in volume (%)	Reasons for neoadjuvant chemotherapy	Outcome
1	77	IV	8.5	8.6	+ 0.1	SD	–	–	–	–	Intravenous tumor thrombus and distant metastasis	Death
2	36	IV	13.5	10.5	− 2.0	SD	868.4	575.0	− 293.4	33.8	Maximum diameter greater than 10 cm and intravenous tumor thrombus	Death
3	113	II	10.6	8	− 2.6	SD	619.0	206.5	− 412.5	66.6	Maximum diameter greater than 10 cm	Alive
4	4	IV	7.0	3.6	− 3.4	PR	55.7	6.6	− 49.1	88.2	Distant metastasis	Alive
5	43	III	11.5	6.7	− 4.8	PR	365.5	94.9	− 270.6	74.0	Maximum diameter greater than 10 cm	Alive
6	59	III	15	13.6	− 1.4	SD	–	–	–	–	Maximum diameter greater than 10 cm and invasion of surrounding tissues	Death
7	12	III	8	7	− 1.0	SD	199.0	116.0	− 83.0	41.7	Invasion of surrounding tissues	Alive

### Outcome

**Fig. 2:**
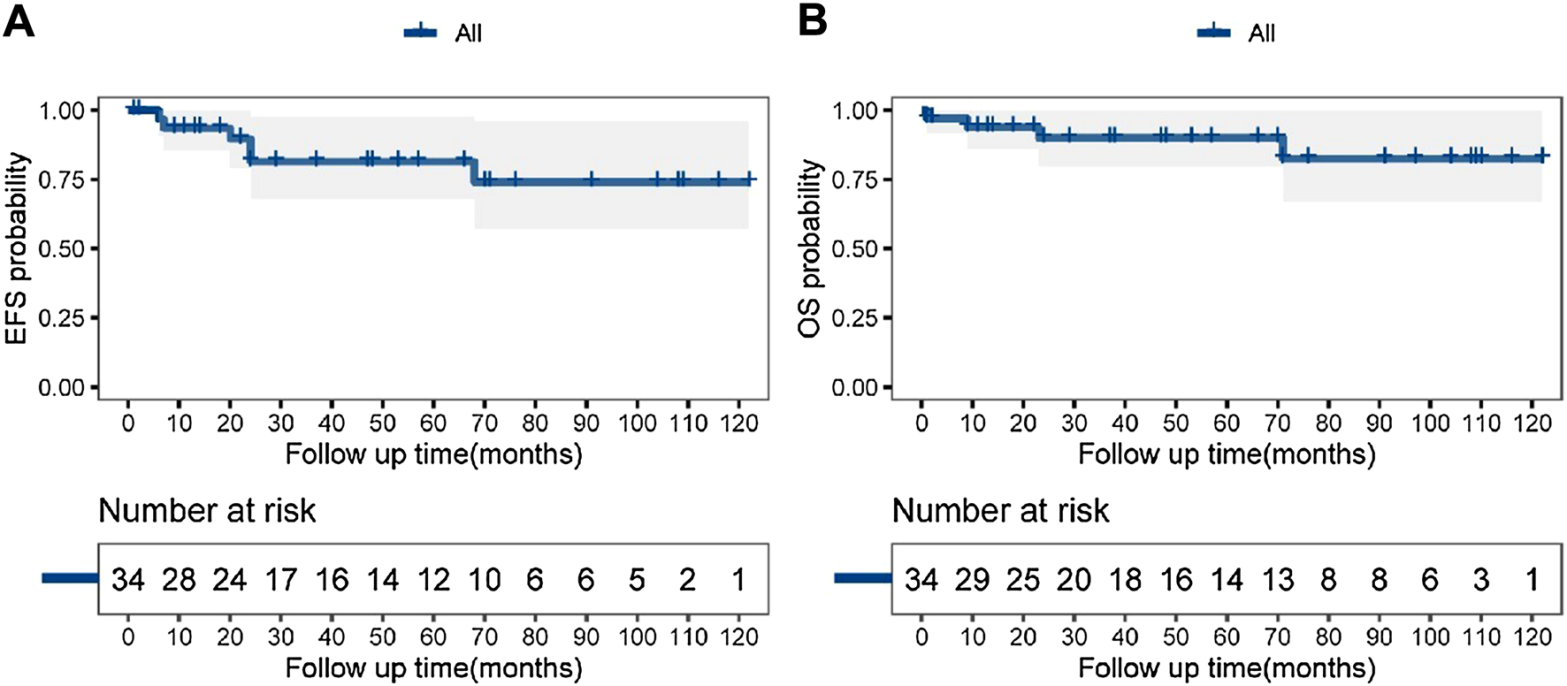
Prognosis of ACC patients. EFS (**A**) and OS (**B**) of the 40 children with ACC

After a median follow-up period of 53 (95% CI-confidence interval 37–91) months, 30 patients (75.0%) remained alive, 4 patients (10.0%) died, and 6 patients (15.0%) were lost to follow-up (all presented with tumor-free survival prior to being lost). Six patients experienced relapse or progression (tumor recurrence in situ, *n* = 3; distant metastasis, *n* = 3). The 5-year OS rate for all children was 90.0% (95% CI 80.0–100.0%), and the 5 year EFS rate was 81.5% (95% CI 68.0–97.7%) (Fig. 2). Among patients who received neoadjuvant chemotherapy, 1/3 patients with stage IV disease, 2/3 with stage III and 1/1 with stage II survived.

## Discussion

ACC is a rare primary adrenal endocrine malignancy associated with germline TP53 pathogenic variant [11, 12], and optimal treatment strategies have yet to be determined. In our study, we summarized the clinical data of 40 pediatric ACC patients and preliminarily explored the indications for and efficacy of neoadjuvant chemotherapy.

Adrenocortical tumors can autonomously secrete excessive amounts of adrenocortical hormones, each of which is associated with specific clinical syndromes [13, 14]. In our study, 14 (35%) patients were newly diagnosed with sexual precocity, 12 (30%) with Cushing’s syndrome, and 5 (12.5%) with virilization. Some studies have reported that there is a correlation between endocrine phenotype and tumor stage. In a retrospective study of 77 ACC patients, Cushing’s syndrome and hypertension were more likely to be found in stage IV disease [5]. In our study, among the 17 children without hormone-related clinical manifestations, 11 patients had abnormal hormone levels at the initial diagnosis, suggesting a time window between the onset of functional tumors and the appearance of related clinical manifestations. We also found that a small number of children had elevated adrenocorticotropic hormone (ACTH), luteinizing hormone (LH), follicle-stimulating hormone (FSH), and prolactin (PRL) hormones, which are rare in ACC. However, most were only slightly elevated and were considered to be disorders of the hypothalamic–pituitary–gonadal axis due to disturbances in sex hormone levels; they can be analogous to the polycystic ovary syndrome in women [15–17]. There were 3 cases of elevated LH, FSH, and PRL in our study, and they were all female, aged 7 months, 3 months, and 13 years old, respectively. Causes of the abnormalities may include the 2 younger children being in mini-puberty, and physiological elevation of hormone levels during puberty in the 13-year-old. Regardless of whether clinical manifestations are present, endocrine levels should be tested comprehensively to facilitate clinical diagnosis and treatment evaluation.

Surgery is still the most effective treatment for ACC. Complete tumor resection can directly affect prognosis, especially for patients without distant metastasis [18–20]. According to the Children’s Oncology Group (COG) ARAR0332 study, radical surgery is the first choice for children with COG stage I/II disease [5]. Previous studies have also confirmed the benefit of repeated surgical resection for recurrent lesions [4, 21]. In our study, 30 children with ENSAT stage I/II disease underwent complete resection; 24 survived without disease, 5 were lost to follow-up, and 1 died (parental refusal due to children poor condition). Postoperative chemotherapy is another important treatment strategy. A regimen of mitotane alone or in combination with EDP is currently the recommended first-line chemotherapy [5, 22, 23]. In our study, 6 of the 16 patients with ENSAT stage II disease were treated with postoperative chemotherapy, and all survived without tumor progression or recurrence. Notably, the survival rate of patients with stage II was 100% in our cohort, which a satisfactory result. In addition to stage III and IV patients, application of postoperative chemotherapy may also be recommended for stage II patients to improve prognosis. Prospective clinical trials are required for further validation.

Surgery and postoperative chemotherapy can significantly improve the prognosis of ACC, but some patients are unfit for surgery at the time of diagnosis due to various conditions. In such cases, neoadjuvant chemotherapy may be an alternative. A 2021 consensus has recently emerged in the pediatric field [13], stating that neoadjuvant chemotherapy can be recommended in children with inoperable and metastatic tumors. However, the definition of “inoperable” has neither been clearly defined nor is evidence-based. Neoadjuvant chemotherapy is rarely used in children with ACC at present. A previous study proposed that “borderline resectability”, the risk of incomplete resection or recurrence, in adult ACC is unacceptable, and immediate surgery is not recommended for these patients [24]. The study divided 53 adults with ACC into 2 groups according to whether complete resection was possible and found that the 5-year OS in the neoadjuvant chemotherapy group was higher than that in the direct surgery group. It was concluded that neoadjuvant chemotherapy was beneficial for these patients [24]. Another study looked at 72 advanced ACC adult patients at first diagnosis who had not been amenable to radical surgery, and were treated with palliative chemotherapy; among them, 5 patients achieved complete remission (CR) and 30 achieved partial response (PR), with an overall response rate of 48.6%. Furthermore, 10 patients underwent radical surgical resection of residual disease after chemotherapy and achieved a disease-free status (13.9%) [25]. To some extent, palliative chemotherapy for advanced ACC confirms the effectiveness of neoadjuvant chemotherapy. Taking into consideration the conclusions of our study and previous studies, we believe that neoadjuvant chemotherapy should be attempted in the following situations for ACC patients: first, a large tumor (largest diameter > 10 cm) that invades the surrounding important blood vessels and tissues; second, preoperative evaluation shows the presence of distant metastases or intravenous tumor thrombus; and third, the patient’s general condition is poor (e.g., cachexia or pulmonary embolism) and precludes surgery. Patients who meet one of these criteria may be eligible to try neoadjuvant chemotherapy. Final treatment decisions should only be made after a multidisciplinary tumor board meeting (MDT) that includes medical oncology, surgical oncology, pediatrics, intensive care unit, and anesthesiology. Benefits of neoadjuvant chemotherapy in children with ACC mainly include the following points. First, reducing the tumor volume can reduce the tumor burden and maximize the possibility of R0 resection. Second, neoadjuvant chemotherapy may help to reduce hormone secretion and ameliorate symptoms of hormonal abnormalities (hormone levels dropped to normal after 3 cycles of neoadjuvant chemotherapy in one of our cases). Third, for children with conditions precluding surgery such as pulmonary embolism or poor nutritional status, neoadjuvant chemotherapy can create an opportunity to resolve correctable complications and thus allow surgery. Fourth, response evaluation during neoadjuvant chemotherapy can help to guide the formulation and adjustment of postoperative chemotherapy regimens. Fifth, chemotherapy may reduce micro-metastasis in particular in case of extend tumor and reduce the risk of distant tumor spread. Based on the above benefits, and used carefully under the guidance of MDT, we expect neoadjuvant chemotherapy to become another important aspect of the treatment strategy for certain children with ACC, especially in stage III and IV disease.

Regarding the better prognosis that was achieved in our study, in addition to the role of neoadjuvant chemotherapy, other conducive factors include high percentage of children with stage I/II (*n* = 30/40) and postoperative chemotherapy that some with stage II underwent. Furthermore, using the Weiss system grading instead of the Wieneke score may have also attributed to better prognosis [26, 27]. Although the Weiss score has a higher sensitivity, its lower specificity might have allowed the more biologically benign ACCs to be treated as malignant. However, confirmation is required via the joint efforts of pathologists and clinicians through prospective trials.

There are some limitations in our study. This was a retrospective study spanning 15 years, some children were lost to follow-up, and the pathologic diagnosis of the children in our study relied on the Weiss score rather than the Wieneke score [19, 28]. Due to the rarity of ACC, the sample size of this study is small, and only 7 patients were treated with neoadjuvant chemotherapy. The specific role of neoadjuvant chemotherapy remains unclear and needs to be demonstrated in prospective randomized controlled trials. A definitive recommendation on the indications for neoadjuvant chemotherapy still cannot be made. Furthermore, mitotane was not used in patients in this cohort because it has not been approved for marketing in China and is difficult to obtain. Pediatric patients should be included in prospective trials in order to define the exact role of medical therapy in such very rare tumor.

In conclusion, for children with ACC, a comprehensive endocrine evaluation is necessary during the treatment process. Early surgical resection should be performed to ensure complete tumor removal, and neoadjuvant chemotherapy can be attempted for certain children to create an opportunity for radical surgery and improve their prognosis.

## Data Availability

The dataset supporting the conclusions of this article is included within the article. The datasets used or analyzed during the current study are available from the corresponding author on reasonable request.
